# Modeling the Fitness Consequences of a Cyanophage-Encoded Photosynthesis Gene

**DOI:** 10.1371/journal.pone.0003550

**Published:** 2008-10-29

**Authors:** Jason G. Bragg, Sallie W. Chisholm

**Affiliations:** 1 Department of Civil and Environmental Engineering, Massachusetts Institute of Technology, Cambridge, Massachusetts, United States of America; 2 Department of Biology, Massachusetts Institute of Technology, Cambridge, Massachusetts, United States of America; University of Warwick, United Kingdom

## Abstract

**Background:**

Phages infecting marine picocyanobacteria often carry a *psbA* gene, which encodes a homolog to the photosynthetic reaction center protein, D1. Host encoded D1 decays during phage infection in the light. Phage encoded D1 may help to maintain photosynthesis during the lytic cycle, which in turn could bolster the production of deoxynucleoside triphosphates (dNTPs) for phage genome replication.

**Methodology / Principal Findings:**

To explore the consequences to a phage of encoding and expressing *psbA*, we derive a simple model of infection for a cyanophage/host pair — cyanophage P-SSP7 and *Prochlorococcus* MED4— for which pertinent laboratory data are available. We first use the model to describe phage genome replication and the kinetics of *psbA* expression by host and phage. We then examine the contribution of phage *psbA* expression to phage genome replication under constant low irradiance (25 µE m^−2^ s^−1^). We predict that while phage *psbA* expression could lead to an increase in the number of phage genomes produced during a lytic cycle of between 2.5 and 4.5% (depending on parameter values), this advantage can be nearly negated by the cost of *psbA* in elongating the phage genome. Under higher irradiance conditions that promote D1 degradation, however, phage *psbA* confers a greater advantage to phage genome replication.

**Conclusions / Significance:**

These analyses illustrate how *psbA* may benefit phage in the dynamic ocean surface mixed layer.

## Introduction

The marine picocyanobacteria *Prochlorococcus* and *Synechococcus* are numerically dominant phytoplankton in nutrient-poor open ocean ecosystems, and are an important contributor to photosynthesis in the oceans [1]–[3]. They are infected by cyanophages including members of the families Podoviridae, Myoviridae and Siphoviridae [4], which can be abundant in regions where these cells dominate (*e.g.* [5]–[8]). Several genomes of these marine cyanophages have been sequenced, revealing gene content and organization broadly similar to confamilial phages [9]–[11]. For example, cyanophage P-SSP7, which infects *Prochlorococcus* MED4, has many genomic similarities to the T7 phage that infects *Escherichia coli* [11].

The genomes of marine *Synechococcus* and *Prochlorococcus* cyanophages often contain genes that are absent from the genomes of morphologically related phages that do not infect marine cyanobacteria [11]. A striking example of this is the *psbA* photosynthesis gene [12], [13]. This gene is found in the genomes of a large proportion of cyanophages known to infect marine picocyanobacteria [14], [15], suggesting that it confers a fitness advantage. The product of the *psbA* gene in the host cell, the D1 protein, forms part of the photosystem II reaction center, and turns over relatively rapidly during photosynthesis [16]. Over the course of phage infection, host-encoded D1 proteins decline following the inhibition of host transcription and the decay of host *psbA* transcripts [17], while phage-encoded D1 proteins increase [17]. It is hypothesized that the latter replace damaged host D1 proteins, and help to maintain photosynthesis throughout the lytic cycle. This, in turn, could increase the relative fitness of phage that carry the *psbA* gene [12], [18]. In some cyanophages, reproduction (*e.g.* [19]) and genome replication [17] are severely limited in the dark, indicating that photosynthesis can be important for phage genome replication, which potentially limits the production of phage progeny. During the cyanophage P-SSP7 lytic cycle, *psbA* is transcribed contemporaneously with several metabolism genes that have probable roles in dNTP synthesis (*e.g.* ribonucleotide reductase), as well as genome replication enzymes [20]. This adds weight to the suggestion that the *psbA* gene helps phage P-SSP7 to acquire resources to make dNTPs during infection.

Models of phage infection in well established phage/host systems, such as T7/*E. coli* [21], have provided significant insights into factors affecting phage reproduction and fitness [22]–[24]. Inspired by these works, we have developed an intracellular model of infection of *Prochlorococcus* MED4 by the Podovirus P-SSP7. The model concentrates on processes of phage genome replication, the production of dNTPs, and the expression of *psbA* by host and phage, and can find good agreement with experimental measurements collected over the cyanophage P-SSP7 lytic cycle [17], [20]. We use the model to ask basic questions about the advantages to the phage of carrying this gene that are not yet tractable experimentally: How much can phage *psbA* expression benefit phage genome replication? To what degree is this contingent on environmental conditions, particularly the ambient light environment?

## Methods

### Model Development

#### *(a)* Approach

**Figure 1:**
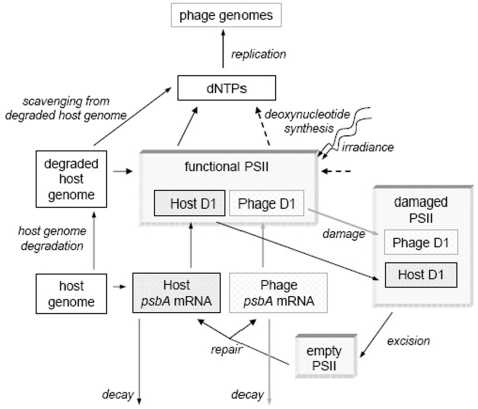
Schematic diagram of model. Phage genomes are made using dNTPs from two possible sources. First, dNTPs can be made by scavenging deoxynucleotides from the host genome. This process can occur in the dark, but is bolstered by photosynthesis. Second, dNTPs can be newly synthesized by a process that is dependent on the products of photosynthesis (dashed lines). Photosynthesis is dependent on functional PSII subunits, which contain the D1 protein. During exposure to light, D1 proteins can become damaged, and are excised from PSII subunits, and replaced with D1 proteins from either host or phage encoded *psbA* mRNAs.

After the genome of cyanophage P-SSP7 enters a host cell, phage genes are expressed, the phage genome is replicated, new phage particles are assembled, and the host cell is lysed — all over a period of about 8 hours [20]. Many of these processes are carried out using products of phage-encoded genes, which are expressed at different times during the cycle of infection [20]. Our model links phage genome replication to the production of deoxynucleoside triphosphates (dNTPs), which in turn is linked to photosynthesis and the kinetics of host and phage *psbA* expression (Fig. 1). It incorporates elements of previous models of phage genome replication [21], [25], and D1 protein kinetics [26]. More specifically, we model phage genome replication within a host cell as a function of the availability of dNTPs, which are supplied by (i) scavenging from the degraded host genome, and (ii) a pathway for the synthesis of new deoxynucleotides. We assume that the supply of dNTPs from each of these sources can depend on photosynthesis. In turn, photosynthesis is modeled as a function of the number of functional photosystem II (PSII) subunits, which become non-functional when their D1 core proteins are damaged, and regain their function when the damaged D1 is excised from the photosystem and replaced with the protein product of either a host or phage *psbA* gene (Fig. 1).

Our model incorporates processes that are carried out by phage genes that begin to be expressed at different times following infection [20]. We therefore need a way to represent relatively abrupt increases in the velocity of processes that are carried out by different proteins (generically, *P*), at different, specific times following infection. We do this using Hill functions [27], or 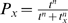, where *t* is the time since infection, *t_x_* is the time at which process *P_x_* reaches half its maximum rate (the ‘timing parameter’), and *n* is a parameter that controls the abruptness of the increase. The Hill function is a sigmoidal curve that increases from zero to one with increasing values of *t*, and can provide a reasonable description of the expression of some relevant phage genes, at least in terms of mRNA abundances (see Text S1). Below, we represent Hill functions in our equations using ‘*P*’ followed by a subscript that corresponds to the process that is being represented.

#### *(b)* Phage genome replication

We assume that protein products of phage genes, such as DNA polymerase, are essential for phage genome replication. Following the expression of these genes, genome replication occurs as a function of the availability of dNTPs, *N* (Fig. 1). We model the change in phage genomes in a host cell (*G_P_*) over time (following [21], [25]), as
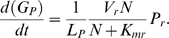
(1)


Here *L_P_* is the length of the phage genome (in base pairs). The term *P_r_* is a Hill function that represents the time-dependent expression of phage genome replication genes. *V_r_* represents the maximum rate of DNA elongation per functional unit of phage genome replication machinery (hereafter, polymerase), multiplied by the maximum abundance of polymerases. *K_mr_* is the value of *N* at which elongation by a polymerase reaches half its maximum rate. We set *G_P_*(0) = 1, to reflect infection by a single virion.

#### *(c)* Phage acquisition of dNTPs

We assume that cyanophage P-SSP7 can acquire dNTPs from two possible sources during infection of *Prochlorococcus* MED4 (Fig. 1). The first is scavenging from the host genome, which is degraded during infection [20]. The second involves the synthesis of new deoxynucleotides [11].

We model degradation of the host's genome (*G_H_*) as
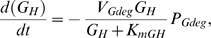
(2)where *P_Gdeg_* is a Hill function representing the expression of genes that degrade the host genome (with time parameter *t_Gdeg_*), and *V_Gdeg_* is the maximum rate of degradation of the host genome. The term 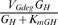, with small *K_mGH_*, is approximately equal to *V_Gdeg_* until the host genome is almost entirely degraded. This formulation is based on the observation that the decline in host genomes is approximately linear, following a delay of 4–5 h [20], as well as the necessity for degradation to cease as *G_H_* approaches 0. We set *G_H_*(0) = 1 to reflect a single host genome at the time of infection.

We assume the phage can then make dNTPs from the degraded host genome (*G_Hdeg_*). We model the rate of production of dNTPs from this source (*s_G_*; dNTPs cell^−1^ h^−1^) as 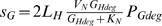. Here 2*L_H_* is the number of deoxynucleotides in the host genome (2 per base pair, times *L_H_* base pairs per genome), and *V_N_* is the maximum rate at which the degraded genome can be converted to dNTPs. We set the parameter *K_N_* to a small value so that when genes for degrading the host genome are expressed and degraded host genome is available, the term 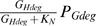 is approximately equal to 1, and dNTPs are produced from degraded genomes at a rate of approximately 2*L_H_V_N_*.

We then consider the possibility that the production of dNTPs from degraded genomes (2*L_H_V_N_*) is limited by photosynthesis. We represent this in the model by letting 2*L_H_V_N_* = *ε*+*μ*, where *ε* represents the rate at which dNTPs can be made from degraded genomes during infection in the dark, and *μ* represents the photosynthesis-dependent production of dNTPs from host genomes. In turn, we assume that *μ* is limited by the abundance of functional photosystem II subunits or *μ* = *zγF_PSII_*, where *F_PSII_* is the number of functional photosystem II subunits per cell, *γ* is the rate of photosynthesis per functional PSII, and *z* is the efficiency with which products of photosynthesis are used in converting degraded genomes to dNTPs. Below, we develop a model for the proportion of PSII subunits that are functional (*f_PSII_*) during infection. We therefore let *F_PSII_* = *Uf_PSII_*, where *U* is the total number of PSII subunits per cell. This means we have *μ* = *zγUf_PSII_*, or if we represent *zγU* by the parameter *κ* (in dNTPs cell^−1^ h^−1^), *μ* = *κf_PSII_*.

We note that the change over time in the proportion of the host genome in a degraded state is then given by
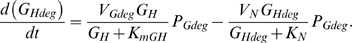
(3)


We now consider the possibility that new deoxynucleotides (*i.e.*, not from the host genome) are produced during infection as a source of dNTPs for phage genome replication (*s_P_*; dNTPs cell^−1^ h^−1^). This possibility is suggested by the observation that cyanophage P-SSP7 encodes [11] and transcribes [20] a ribonucleotide reductase gene, whose protein product likely functions in converting ribonucleotides to deoxynucleotides. We assume this source of dNTPs is dependent on photosynthesis, as well as the activity of genes that are encoded by the phage, and whose expression is described by a Hill function, *P_S_*. Once these phage genes are expressed, the rate of supply of dNTPs from this source is assumed to be proportional to the rate of photosynthesis, which is limited by the abundance of functional PSII subunits, or *s_P_* = *vγF_PSII_P_S_*, where *v* is the efficiency with which products of photosynthesis are converted to dNTPs. We then represent *vγU* using a single parameter, *λ* (in dNTPs cell^−1^ h^−1^), such that *s_P_* = *λf_PSII_P_S_*. Presently we lack detailed mechanistic information about this potential extra source of dNTPs, which would be useful for refining the model. For example, if photosynthesis powers the conversion of a finite cellular resource to dNTPs, the depletion of this resource ought to be modeled.

After accounting for the incorporation of free dNTPs into genomes, we have a rate equation for dNTPs per cell (*N*):
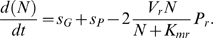
(4)


We next model the proportion of PSII subunits that are functional (*f_PSII_*), to insert in both *s_G_* and *s_P_*. Functional PSII subunits are lost when their D1 proteins become damaged. Following excision of the damaged D1 protein, PSII subunits become functional upon receiving a new D1 protein. Our approach is similar to that of [26], in modeling the proportions of PSII subunits that (i) are functional (*f_PSII_*), (ii) contain damaged D1 proteins (*d_PSII_*), and (iii) have had damaged D1 proteins excised (‘empty’ PSII subunits, or *x_PSII_*) (Fig. 1). For functional and damaged subunits, we track PSII subunits containing host- versus phage-encoded D1 proteins separately. For example, for functional PSII subunits, *f_PSII_* = *f_PSIIH_*+*f_PSIIP_*, where *f_PSIIH_* and *f_PSIIP_* contain host D1 and phage D1, respectively. We also assume that during the course of infection, the total number of PSII subunits (*U*) in a cell is constant, and that *f_PSIIH_*+*f_PSIIP_*+*d_PSIIH_*+*d_PSIIP_*+*x_PSII_* = 1. This yields the following system of equations:
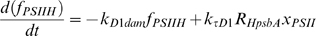
(5)

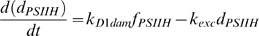
(6)

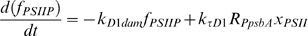
(7)

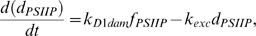
(8)where *x_PSII_* = 1−(*f_PSIIH_*+*f_PSIIP_*+*d_PSIIH_*+*d_PSIIP_*). Here *k_D1dam_* is the rate at which D1 proteins in functional PSII subunits are damaged by irradiance, *k_exc_* is the rate at which damaged D1 proteins are excised from PSII subunits, and *k_τD1_* is the rate at which damaged PSII subunits are repaired using *psbA* mRNA transcripts. *R_HpsbA_* and *R_PpsbA_* are the abundances of host and phage *psbA* transcripts, respectively. This formulation assumes that D1 proteins are represented only in functional and damaged PSII subunits, and that *psbA* transcripts are limiting to repair.

The expression of host and phage *psbA* transcripts are modeled as follows:
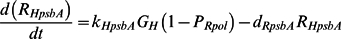
(9)

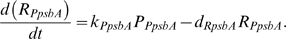
(10)


Here *d_RpsbA_* is the decay rate of *psbA* mRNA transcripts. *k_HpsbA_* and *k_PpsbA_* are the maximum rates of transcription of host and phage *psbA* mRNAs, respectively. Host *psbA* is transcribed until either the host genome is gone, or until host RNA polymerase is inhibited. The inhibition of host RNA polymerase by a phage protein is represented using the term (1-*P_Rpol_*), where *P_Rpol_* is a Hill function. *P_PpsbA_* is a Hill function representing the commencement of transcription of phage *psbA* at a time of approximately *t_PpsbA_*.

The above formulation includes assumptions that can be tested experimentally, and improved in future versions of the model. We assume, for example, that host and phage *psbA* transcripts have identical rates of decay (*d_RpsbA_*). We also assume that empty PSII subunits can be repaired at identical rates using products of host and phage *psbA* genes, that PSII subunits containing host and phage D1 proteins have similar rates of damage (*k_D1dam_*) and excision (*k_exc_*), and that functional PSII subunits containing host and phage D1 have similar rates of photosynthesis. In reality, these properties of host and phage *psbA* transcripts or D1 proteins could be different. For example, it has been suggested that phage D1 might be more resistant to photodamage than host D1 [28]. Furthermore, we assume that the total number of photosystem II subunits and the maximum rates of excision and repair are constant over the course of infection, while in reality, these values may decay as a function of time. It would be useful to measure these properties of infected cells experimentally, and revise the model if necessary. More broadly, our model clearly uses a highly simplified representation of photosynthesis, an extremely complex process influenced by a large number of factors [29]. Our goal was to abstract this complexity with a focus on the potential advantage to phage of supplementing the supply of D1 during infection.

## Results

### Model validation

#### *(a)* Approach

The parameterization of the model is described in detail below, and parameter values are listed in Table 1. Our general approach was as follows: We began by considering parameters that govern the abundance of host and phage *psbA* transcripts, and then estimated parameters for the abundance of host and phage D1 proteins. In the experiments that are the basis for the model, cells were grown under continuous light [17]. We therefore assumed the abundances of host *psbA* mRNAs and the proportions of functional, damaged and empty PSII subunits were in steady state prior to infection, and set equations (5), (6) and (9) equal to 0. This imposed relationships between parameters and initial conditions of some variables (Table 1), reducing the number of free parameters. Finally, we used data for genome replication in the light and dark [17] to estimate parameters for the dependence of dNTP acquisition on photosynthesis. Data from Lindell et al. [20] were used to estimate parameters for the degradation of host genomes and for the timing of expression of phage genes involved in genome replication and dNTP production (see Text S1).

**Table 1:** Model parameters and initial conditions.

Parameter	Description	Units	Value
*G_P_*	phage genomes	genomes cell^−1^	1*
*G_H_*	host genomes	genomes cell^−1^	1*
*G_Hdeg_*	degraded host genomes	genomes cell^−1^	0
*N*	dNTPs	dNTPs cell^−1^	0
*f_PSIIH_*, *f_PSIIP_*	proportion of PSII subunits that are functional and contain host, phage D1	dimensionless	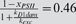 , 0*
*d_PSIIH_*, *d_PSIIP_*	proportion of PSII subunits that are damaged and contain host, phage D1	dimensionless	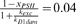 , 0*
*x_PSII_*	proportion of PSII subunits that are empty	dimensionless	0.5*,a
*R_HpsbA_*, *R_PpsbA_*	*psbA* transcripts	dimensionless	1, 0*
*L_H_*, *L_P_*	genome length of host, phage	bp genome^−1^	1657990, 44970
*V_r_*	max velocity of phage DNA elongation	bp h^−1^ cell^−1^	1332000
*K_mr_*	half-saturation for DNA replication	dNTP cell^−1^	1224
*t_r_*	timing parameter for phage genome replication	h	2
*V_Gdeg_*	max velocity of host genome degradation	genomes cell^−1^ h^−1^	0.35
*K_mGH_*	half-saturation for host genome degradation	genomes cell^−1^	0.000001
*t_Gdeg_*	timing parameter for host genome degradation	h	5
*ε*	production of dNTPs from degraded host genome in the dark	dNTP h^−1^ cell^−1^	127665
*κ*	production of dNTPs from degraded host genome in the light	dNTP h^−1^ cell^−1^	0
*K_N_*	half saturation for dNTP production from degraded host genome	genomes cell^−1^	0.000001
*λ*	production of dNTPs in the light	dNTP h^−1^ cell^−1^	1027800
*t_S_*	timing of dNTP synthesis from source *s_P_*	h	4
*k_D1dam_*	damage to functional D1 proteins	h^−1^	0.35^a^
*k_exc_*	excision of damaged D1 proteins	h^−1^	4^a^
*k_τD1_*	repair of empty PSII subunits	h^−1^	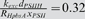
*d_RpsbA_*	*psbA* transcript decay	h^−1^	0.27^b^
*k_HpsbA_*	host *psbA* transcription	h^−1^ (genomes cell^−1^)^−1^	0.27^b^
*k_PpsbA_*	phage *psbA* transcription	h^−1^	0.016
*t_Rpol_*	timing parameter for inhibition of host RNA polymerase	h	1
*t_PpsbA_*	timing parameter for transcription of phage *psbA*	h	1.3
*n*	Hill parameter	dimensionless	5

* Initial condition.

a Values were systematically varied in exploring the kinetics of D1 protein degradation, excision and repair. All combinations of the following values were used: *k_D1dam_* = [0.01, 0.025, 0.05, 0.06, 0.07, 0.08, 0.09, 0.1, 0.125, 0.15, 0.175, 0.2, 0.25, 0.3, 0.35, 0.4, 0.5].

*k_exc_* = [0.5, 0.75, 1, 1.25, 1.5, 1.75, 2, 2.5, 3, 3.5, 4, 4.5, 5, 7.5, 10].

*x_PSII_*(0) = [0.05, 0.1, 0.15, 0.2, 0.25, 0.3, 0.4, 0.5, 0.6, 0.7, 0.8, 0.9].

b This estimate is based on microarray measurements of host *psbA* mRNA expression. Measurements of host *psbA* transcript abundances that were made using RT-PCR [17] suggested a greater value of *d_RpsbA_*. We therefore present additional analyses based on a value of *d_RpsbA_* = 0.72 in Text S1.

Lindell et al. [17], [20] studied populations of cells that were infected with phage, while our model is based on infection of a single host cell. In comparing model predictions to these experimental data, we assume that our model represents infection of an average cell. To estimate the number of phage genomes per host cell at different times after infection, we normalized by the number of phages measured at 1 h post-infection. Given that our estimates of phage genome replication depend on this normalization, we place our emphasis on the proportional advantage or disadvantage conferred by phage *psbA*, rather than the absolute number of genomes. Lindell et al. [17] used a low multiplicity of infection (0.1 phage for every host cell) for the experiment in which phage genome replication was measured. Under these conditions, most infected cells would have been infected by a single virion. When using data for intracellular levels of host *psbA* transcripts, D1 proteins, and genomes, we assumed that 50% of cells were infected (see Text S1 for analyses that consider the implications of varying this assumption). A higher multiplicity of infection (3 phage per host cell) was used in the experiments from which these data were collected, and 50% represents the maximum level of infection that has been observed for this phage [17]. We also assumed that measurements of D1 protein abundance made by [17] detected both functional and damaged D1 proteins.

We integrated equations (1)–(10) using ode45, a MATLAB® (The MathWorks, Natick, MA) variable time step numerical ODE solver, which implements a medium order Runge-Kutta scheme.

#### *(b)* Expression of photosynthesis genes

**Figure 2:**
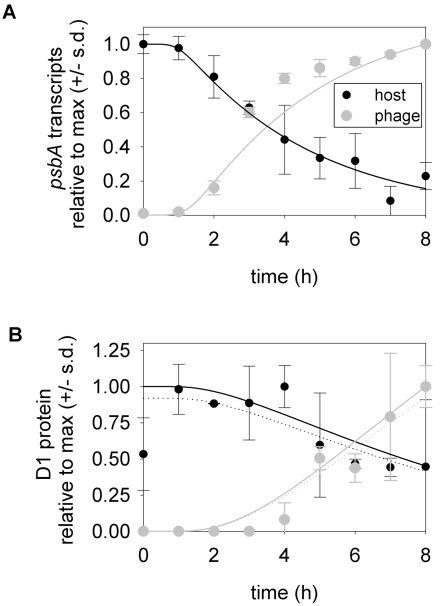
Measured (data points) and modeled (lines) levels of host and phage *psbA* transcripts (A) and D1 protein product (B) during the lytic cycle of infection of *Prochlorococcus* MED4 by cyanophage P-SSP7. For modeled levels of D1 protein, solid lines represent the sum of functional and damaged D1, and dotted lines represent functional D1 only. Data are from [17]. Data for host expression levels were transformed assuming that 50% of cells were infected [17].

Experimental evidence shows that following infection by the cyanophage P-SSP7, the abundance of *psbA* transcripts in the host cell declines [17]. We assumed that host transcription was largely inhibited (1-*P_Rpol_*≈0) by 1 hour after infection (Fig. 2A, Table 1), and calculated the decay constant (*d_RpsbA_*) using experimental observations [17]. We set the initial value of host *psbA* mRNA to *R_HpsbA_*(0) = 1, and normalized the abundance of host *psbA* transcripts to this initial (maximum) value.

Following the decay of host *psbA* transcripts, Lindell et al. [17] observed a drop in the level of host D1 proteins, such that host D1 abundance had decreased to approximately 45% of its maximum value (measured 1 hour after infection) after 8 hours of infection (Fig. 2B). In parameterizing the dynamics of host D1 proteins, we first assumed cells were in steady state prior to infection, which constrained parameters according to *R_HpsbA_*(0)*k_τD_*_1_ *x_PSII_*(0) = *k_exc_* *d_PSIIH_*(0) = *k_D_*_1*dam*_ *f_PSIIH_*(0). This means only *x_PSII_*(0) (and correspondingly, *k_τD1_*; see Table 1) was free to vary for given pair of *k_exc_* and *k_D1dam_* values (since *R_HpsbA_*(0) = 1, and *x_PSII_*(0)+*d_PSIIH_*(0)+*f_PSIIH_*(0) = 1). We did not have independent estimates of the parameters *k_D1dam_*, *k_exc_* and *x_PSII_*(0) for *Prochlorococcus* under the conditions of the experiment [17], and values of *k_D1dam_* and *k_exc_* may vary substantially among organisms and growth conditions [26]. We therefore used measurements from a study of *Prochlorococcus* PSII function and D1 protein abundance under transient exposure to high irradiance [30] to estimate possible ranges of parameters *k_D1dam_*, *k_exc_* and *x_PSII_*(0). We then solved our model of D1 dynamics 3060 times, comprising all combinations of 17 values of *k_D1dam_*, 15 values of *k_exc_*, and 12 values of *x_PSII_*(0) (see Table 1). Out of these 3060 simulations, 126 resulted in a drop in the abundance of host D1 proteins after 8 hours of infection that was similar to the value measured in the laboratory [17]. From here onward, we present analyses that focus on one set of parameters (*k_D1dam_* = 0.35 and *k_exc_* = 4, with *x_PSII_*(0) = 0.5), but we did perform all subsequent analyses using all 126 combinations of parameters, to confirm that our conclusions are robust across this range of parameter values (see Text S1). The model can provide a reasonable description of the drop in host D1 proteins during infection, as illustrated in Fig. 2B (black line and symbols). However, we note that the model does not predict several features of the experimental observations, and in particular, the low level of host D1 at 0 hours, and the sudden drop in host D1 between 4 hours and 5 hours after infection. We are not aware of any mechanisms that might account for these observations, so have not attempted to replicate them with the present model.

We next modeled the abundance of phage *psbA* transcripts using the decay constant (*d_RpsbA_*) calculated above for host *psbA* transcripts. Our model describes the shape of the experimentally derived curve of phage *psbA* mRNA abundance reasonably well (Fig. 2A), though modeled levels of mRNAs approached an asymptotic level more slowly than observed [17].

The empirical observation that phage D1 proteins accumulated to approximately 10% of all D1 after 8 hours of infection [17] was predicted by the model when phage *psbA* transcription was set to 5.9% of the rate at which host *psbA* was transcribed prior to infection (*k_PpsbA_* = 0.016; Fig. 2B), and with the same values of *k_D1dam_* and *k_exc_* that were used for host D1. The model predicted the increase in the level of phage D1 slightly sooner than it was observed experimentally. This could be due to a time delay for the translation of D1, or may simply reflect experimental variability.

#### *(c)* Degradation of the host genome

Experimental evidence showed that host genomes are mostly degraded between 4 and 8 hours after infection [20] and the loss of host genomes was approximately linear. The model provides a good description of these observations with *t_Gdeg_* = 5 and *V_Gdeg_* = 0.35, and with *K_mGH_* set to a small value (0.000001) (data not shown).

#### *(d)* Genome replication

**Figure 3:**
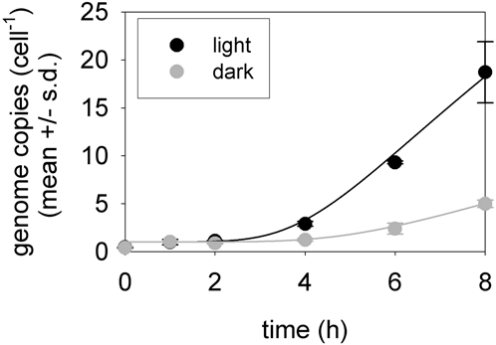
Measured (data points) and modeled (lines) genome copies of cyanophage P-SSP7 during the lytic cycle under light (25 µE m^−2^ s^−1^) and dark conditions. Genome copies were measured as genomes per ml of culture [17], and were transformed to a per cell basis for comparison to the model.

To study phage genome replication in the model, we first simulate infection in the dark, setting the photosynthesis-dependent production of dNTPs equal to zero (setting *λ* = 0 and *κ* = 0). We then needed to estimate values for DNA replication kinetic parameters (*V_r_* and *K_mr_*), the timing of phage DNA replication machinery (*t_r_*) and the production of dNTPs using degraded host genomes in the absence of photosynthesis (*ε*). Phage T7 has a rate of DNA elongation of approximately 1,332,000 (h^−1^ polymerase^−1^) [21], [31]. In the absence of data for cyanophage P-SSP7, we set *V_r_* = 1,332,000 (bp h^−1^ cell^−1^). We did not multiply this value by the number of phage polymerases in the host cells since (i) we do not have data on phage polymerase abundance, and (ii) this value of *V_r_* is already sufficiently large to be non-limiting to genome replication (see below). We also estimated *K_mr_* based on the corresponding value for deoxynucleotide incorporation by T7 phage enzymes [21], [32], adjusted according to the size of a *Prochlorococcus* MED4 cell, which is assumed to be a sphere with diameter 0.6 *µ*m. We assumed that phage genome replication enzymes were expressed approximately 2 hours post-infection (*t_r_* = 2), based on observations of phage DNA polymerase transcript abundance in [20] (see Text S1). We then found that *ε* = 127,665 could provide a reasonable description of genome replication in the dark, if all dNTPs used in phage genome replication in the dark were derived from the host genome (Fig. 3).

Our model includes the possibility that photosynthesis increases the production of dNTPs from degraded host genomes, and the possibility that photosynthesis promotes the synthesis of new deoxynucleotides. However, since we do not know the relative importance of these possible sources of dNTPs, we analyze their potential contribution to dNTP production during infection in the light separately. Here we present analyses that assume extra dNTPs made in the light were derived from the synthesis of new deoxynucleotides (i.e., *λ*>0 and *μ* = 0). However, we confirmed that similar results are obtained if we assume instead that the extra dNTPs made in the light were derived from the degraded host genome (see Text S1).

For infection in the light, we use the same values of parameters *V_r_*, *K_mr_*, *t_r_* and *ε*, and found that *λ* = 1,027,800 (dNTP h^−1^ cell^−1^) gave a reasonable description of phage genome replication (Fig. 3). With this parameterization, dNTP availability is strongly limiting to genome replication: a 10% increase in dNTP production by photosynthesis (*λ*) results in a 7.3% increase in genome replication, whereas a 10% increase in the maximum velocity of genome replication (*V_r_*) results in almost no increase in genome replication.

### *In silico* knockout of phage *psbA*

The major goal of this study is to consider the fitness consequences to a phage of encoding and expressing the *psbA* gene. Having described the kinetics of infection reasonably well with our model (Figs 2 and 3), we can now turn off transcription of phage *psbA* (*k_PpsbA_* = 0), and study how this affects the predicted number of phage genomes in infected cells after 8 h of infection. Using the parameter values presented in Table 1, we predict that a phage unable to express *psbA* would produce 2.81% fewer genomes after 8 h of infection. However, if a phage did not encode *psbA*, its genome would be shorter, by approximately 1080 bp. Taking this into account, a phage that did not encode *psbA* would produce only 0.55% fewer genomes after 8 h of infection than a phage that encodes and expresses *psbA*. In the dark, where there is presumably no advantage to expressing *psbA*, a phage without this gene is predicted to produce 1.97% more genomes than a phage with it.

Ideally, we would like to consider the consequences of *psbA* to phage genome replication under different and changing levels of irradiance. However, many of the parameters used in our model are likely to change as a function of irradiance, in ways that can be difficult to predict (see [29]). Therefore we limit ourselves to one specific case, where cells are moved from 25 µE m^−2^ s^−1^ to 50 µE m^−2^ s^−1^ one hour after infection has begun, when the capacity of the cells to respond to the changing light may be largely compromised by infection. This means we can use the same initial conditions and parameter values as in our previous simulations (Table 1), except for two parameters that will be affected directly by the increased irradiance (*k_D1dam_* and *λ*). We assume that the rate of damage to functional PSII subunits (*k_D1dam_*) increases proportionally with irradiance (i.e., *k_D1dam_* is doubled; [26]), and that the rate of photosynthesis of *Prochlorococcus* MED4 is greater at 50 µE m^−2^ s^−1^ than at 25 µE m^−2^ s^−1^ by a factor of approximately 1.75 [33], [34].

We found that in the case where irradiance increases from 25 µE m^−2^ s^−1^ to 50 µE m^−2^ s^−1^ one hour after infection, a phage that does not express *psbA* is predicted to produce 4.31% fewer genomes than a phage that does. Here, a phage that does not express or encode *psbA* is predicted to produce 2.10% fewer genomes than a phage that does encode and express *psbA*. We therefore predict that *psbA* will have a greater impact on phage genome replication under this switch to a higher level of irradiance, such as could occur in the surface mixed layer of the oceans. We note that this prediction also holds if photosynthesis (and *λ*) increases by a factor of either 1.5 or 2 under the switch to higher irradiance, rather than by a factor of 1.75 (see Text S1). Further, we performed analyses similar to the above using a range of different values of *k_D1dam_*, *k_exc_* and *x_PSII_*(0). Across these simulations, expressing *psbA* usually led to a modest increase in genome replication in continuous light (of between 2.5 and 4.5%), though this increase was typically smaller (between 0.3 and 2.3%) when the cost of encoding *psbA* was considered. The predicted advantage of expressing and encoding *psbA* was typically greater when a switch to higher light was simulated during infection, though the precise size of the advantage conferred by *psbA* varied (see Text S1).

### Cyanophage dNTP diets

In addition to *psbA*, marine cyanophages encode a variety of genes that potentially help them acquire dNTPs (*e.g.*, ribonucleotide reductase, transaldolase; see [35]). It is therefore interesting to ask more broadly: Under what set of circumstances can a phage increase its total genome replication by encoding an additional gene or module of genes that help it acquire extra dNTPs?

Consider a phage that encodes genes that allow it to access dNTPs from a single source, *s_1_* (*e.g.* scavenging from the host genome). Now, a mutant acquires an extra gene or module of genes that allow it to access an extra source of dNTPs, *s_2_*. If genes needed to access this second source of dNTPs elongate the wild type genome, we want to know when the mutant will make more genomes than the wild type by some time post-infection, or when
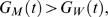
(10)where *G_M_*(*t*) and *G_W_*(*t*) are the numbers of genomes in cells infected by mutant and wild type phages (respectively) at time *t*.

We explore this question using our original model as a starting point, but with modifications that allow it to be studied analytically. We assume that the supply of dNTPs is highly limiting to genome replication (*N*≪*K_mr_*), such that we can rewrite equation (1) as 
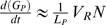
, where *V_R_* is the rate of DNA elongation (here in bp dNTP^−1^ h^−1^ cell^−1^). We also assume that the dNTP revenue from each phage-encoded source can be expressed as a function of time, such that a phage using *s_1_* and *s_2_* has 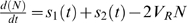. In reality, processes of phage-encoded dNTP acquisition (*s_1_* and *s_2_*) and genome replication may begin at different times post-infection. For simplicity, we assume these processes all begin at the same time (*t_b_* hours after infection), and let time *t* = 0 in this model refer to this time *t_b_* when these processes begin. Solving for *N*(*t*), and then *G_P_*(*t*) yields
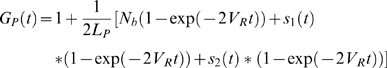
(11)where *N_b_* is the number of dNTPs in the cell at time *t_b_*, there is one phage genome in the cell at time *t_b_*, and ‘*’ represents a convolution product. If we assume *N_b_* is very small (*N_b_*≈0) and sub (11) into (10), we get
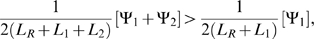
(12)where Ψ_1_ and Ψ_2_ represent dNTPs derived from *s_1_* and *s_2_* (respectively), *L_1_* and *L_2_* represent the length of genes needed to encode *s_1_* and *s_2_* (respectively) and *L_R_* represents the length of the rest of the genome. This can be expressed as 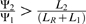, meaning that a new module of genes will increase phage genome replication if it leads to a proportional increase in dNTP production that is greater than the proportional increase in genome length that it causes. Alternatively, we could say that for a new module of genes to increase phage genome replication, it must have a ratio of dNTPs contributed / cost in genome length, 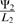, that exceeds a threshold, 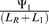.

While this model is oversimplified, and has required assumptions that limit its applicability, it nevertheless may help us to understand some of the variability among cyanophages in methods they use to acquire dNTPs. For example, it suggests that if two similar phages acquire dNTPs by scavenging from the genomes of their hosts (i.e., their *s_1_*), but one phage infects a host with a smaller genome from which fewer dNTPs can be produced (smaller Ψ_1_), this phage might be more likely to exploit an additional source of dNTPs, if given the opportunity. This may be one factor that helps to explain why cyanophages infecting *Prochlorococcus*, which has a very small genome, might encode genes that help acquire dNTPs from other sources (see [36] for discussion of related issues).

Further, it can be shown that if the new source of dNTPs, *s_2_*, is highly profitable, the phage may no longer be advantaged by encoding *s_1_*. We would expect this to be the case when 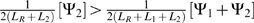, or when 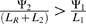. This illustrates a way in which one source of dNTPs could replace another in the genome of a phage, over evolutionary time.

This analysis has strong parallels with diet theory models that predict when a foraging animal should incorporate an encountered prey item into its diet, based on the energetic gain from the prey item, balanced against the cost in terms of time of pursuing it [37], [38]. It thus adds to an impressive list of circumstances in which phage strategies can be understood using analogies to theory developed for foraging animals (*e.g.* [39], [40]).

## Discussion

The goal of this simple modeling exercise was to predict the advantage conferred to a cyanophage of carrying and expressing the *psbA* gene. More specifically, we consider the hypothesis that phage *psbA* expression augments the photosynthetic apparatus of the host during infection, following the decay of host *psbA* transcripts, and we do not consider possible alternative or additional advantages of phage *psbA*. We have intentionally oversimplified the complex processes of infection, photosynthesis and dNTP synthesis in an effort to match the model to the scope and resolution of the available data. The modeled predictions serve as hypotheses to be tested when the means to knock out specific genes in these cyanophage genomes are eventually developed.

First, we predict that under low continuous irradiance, phage *psbA* expression increases phage genome replication, and potentially phage fitness, relative to a ‘mutant’ that does not contain this gene. This advantage is substantially reduced, however, if one accounts for the cost to the phage of elongation of the cyanophage P-SSP7 genome by *psbA*. Second, we predict that the slight advantage conferred by phage-encoded *psbA* may be greater under conditions of light stress, such as an increase in irradiance during infection. This is due to the more rapid decay of host D1 proteins at higher irradiance, and could contribute substantially to the advantage conferred by *psbA* to cyanophage P-SSP7 in the dominant habitat of this particular *Prochlorococcus* host — the surface mixed layer of the ocean. Finally, the model predicts that during infection in the dark, where there is presumably no advantage to expressing *psbA*, encoding *psbA* would result in a net decrease in genome replication of approximately 2%. Taken together, these results illustrate how the benefits of *psbA* to cyanophage genome replication may vary substantially among infections that occur at different times over the diel cycle, or for cells that are subject to different conditions of irradiance due to mixing [18]. These are all testable hypotheses.

It is clear that the selective advantage of *psbA* to phage will be determined by the benefit it confers during all conditions under which infection occurs, weighted by their frequency of occurrence. Therefore to fully understand the fitness consequences of carrying the *psbA* gene to phage, we will need to better understand how *psbA* influences genome replication over a much broader range of conditions, including at different times over the diel cycle where properties of host photosynthesis will change dynamically [29] and hosts will contain different numbers of genomes [41] and free dNTPs. To connect these predictions for genome replication to fitness, we will also need a better understanding of when genome replication limits phage burst size (*e.g.* see [24], [42], [43]) and of the interactions between burst size and other factors, such as the timing of cell lysis, and the availability and quality of hosts (*e.g.* [39], [40], [44]–[47]).

We have learned recently that marine cyanophage encode a number of genes that are absent in the genomes of non-marine phages and share homology with genes involved in microbial metabolism [11]. As we attempt to understand both the evolutionary significance of these genes and the distribution of phage genes in the ocean [35], it will be useful to have theoretical tools. To begin building such tools, here we have developed a model exploring the selective advantage of one specific gene of host origin that is commonly encoded by marine cyanophages, as well as more general tradeoffs between acquiring dNTPs and elongating the genome. We hope these models will form the basis for a more powerful and predictive modeling framework, and contribute substantially to our understanding of phage dynamics in marine microbial communities.

## Supporting Information

Text S1 Description of simulations using a range of different parameter values.
(0.34 MB PDF)
