# Supplementary material for: Modeling the Fitness Consequences of a Cyanophage-Encoded Photosynthesis Gene
Source: PLoS One. 2008 Oct 29;3(10):e3550. doi: 10.1371/journal.pone.0003550 (PMC2570332; doi:10.1371/journal.pone.0003550)
Supplement: Text S1 — Description of simulations using a range of different parameter values. (0.34 MB PDF) [file pone.0003550.s001.pdf]

Supporting Information: Text S1

We tested the predictions of our model using a range of different values of the rate of damage to D1 (k_{D1dam}), the rate of excision of damaged D1 (k_{exc}) and the proportion of photosystem II subunits that contained no D1 protein at the time of infection ($x_{PSII}(0)$). To determine realistic possible ranges for these parameters, we applied the model of PSII dynamics in equations (5) and (6) (originally developed in ref. [1]) to measurements of D1 protein abundance in *Prochlorococcus* ecotype PCC9511 during a transient increase in irradiance [2]. We determined ranges of k_{exc} and x_{PSII} that could account for the difference in the loss rate of D1 protein between lincomycin treated and untreated cells. We also used measurements in Six et al. [2] to inform the range of values of k_{D1dam} we considered, though this parameter could have varied substantially between high irradiance [2] and phage infection [3] treatments.

The predictions of our model for the proportion of host D1 remaining after 8 h of infection are illustrated in Fig. S1.

In our initial analyses of the model, we used a decay rate for host *psbA* mRNA of d_{RpsbA} = 0.27 (h^{-1}), which was based on microarray measurements of *psbA* mRNA expression [3]. However, we found that this decay rate was greater (d_{RpsbA} = 0.72) when estimated using RT-PCR measurements [3]. We therefore repeated our analyses using a value of d_{RpsbA} = 0.72. Model predictions for the proportion of host D1 remaining after 8 h of infection with d_{RpsbA} = 0.72 are illustrated in Fig. S2. In these analyses, the decay rate of phage *psbA* mRNA is also set to k_{PpsbA} = 0.72, and the commencement of phage *psbA* expression occurs slightly later, with t_{PpsbA} = 1.8 (h). A notable observation from these analyses is that when the decay of *psbA* mRNA is assumed to be high, lower values of

k_{D1dam} can lead to the decay of host D1 protein to levels that are similar to those observed experimentally [3].

Our initial analyses of the model also assumed that 50% of cells were infected in the experimental measurements of *psbA* expression [3]. We repeated our analyses assuming that 63% of cells were infected in the experiment where phage *psbA* expression was measured [3]. This possibility was suggested by the observation that RT-PCR measurements of phage *psbA* transcripts decline to less than 50% of their initial value by the end of the lytic cycle [3]. In Fig. S3, we present model predictions for the proportion of host D1 remaining after 8 h of infection, when we assume that $d_{RpsbA} = 0.72$ and that 63% of cells were infected.

Across the range of parameter value combinations satisfying the experimental measurements of Lindell et al. [3], most predict that a phage which encodes and expresses *psbA* will produce slightly more genomes after 8 h of infection (0 – 2.3%), than a phage that does not express and does not encode *psbA*, but that this advantage of expressing and encoding *psbA* is greater under higher irradiance (Fig. S4). These observations hold if one assumes that all the extra dNTPs acquired for phage genome replication in the light are acquired from the synthesis of new deoxynucleotides, or are produced by scavenging from the host genome (Fig. S5). However, it is important to note that under different conditions of infection, nucleotides from the host genome (or any finite source) could be exhausted, and may not account for all of the dNTPs produced by the phage during infection.

In Fig. S6, we illustrate how model parameters describing the timing of genome replication (t_r) and dNTP synthesis (t_s) correspond to the expression of genes that are likely involved in these processes.

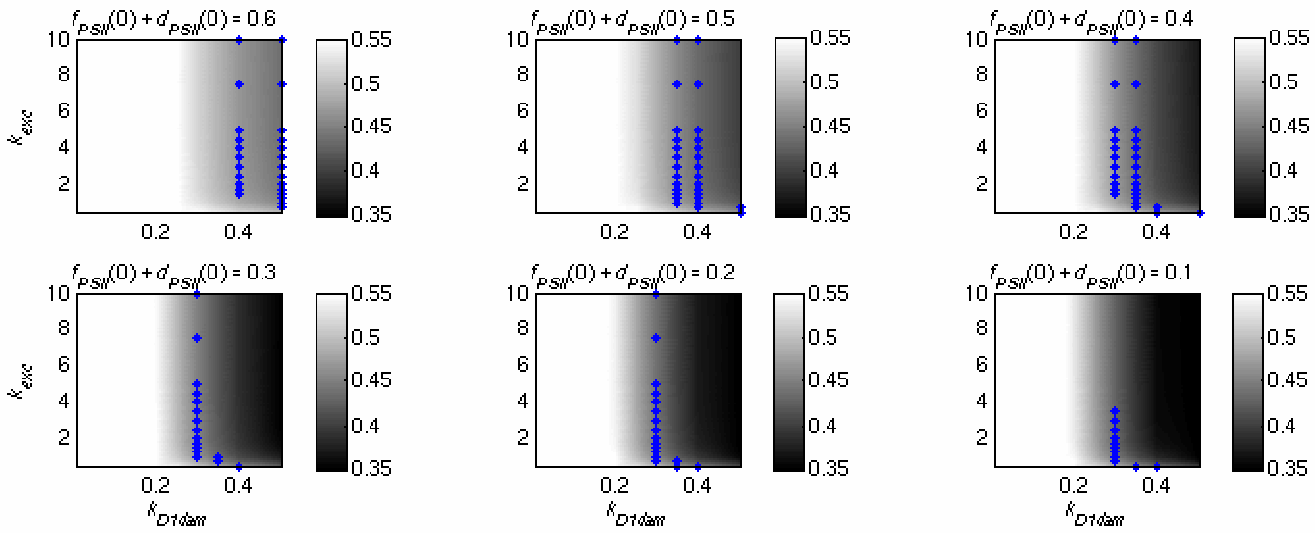

Fig. S1. Model predictions for the proportion of host D1 protein remaining after 8 h of infection, as a function of the decay rate of D1 (k_{D1dam}; horizontal axes), the excision rate of damaged D1 (k_{exc}), and proportion of photosystem II subunits that contained a D1 protein at the time of infection ($f_{PSII}(0)+d_{PSII}(0)$, or $1 - x_{PSII}(0)$; different panels). These solutions assume that $d_{RpsbA} = 0.27$, and that 50% of cells were infected in experiments where phage *psbA* expression was measured [3]. Blue dots illustrate combinations of parameter values that can account for the drop in levels of host D1 protein to 45% of their initial value, following 8 hours of infection (±5%), in the presence of appropriate expression of *psbA* by phage. Values of $f_{PSII}(0)+d_{PSII}(0)$ greater than 0.6 are not shown, since no solutions were found that satisfied experimental observations at these values.

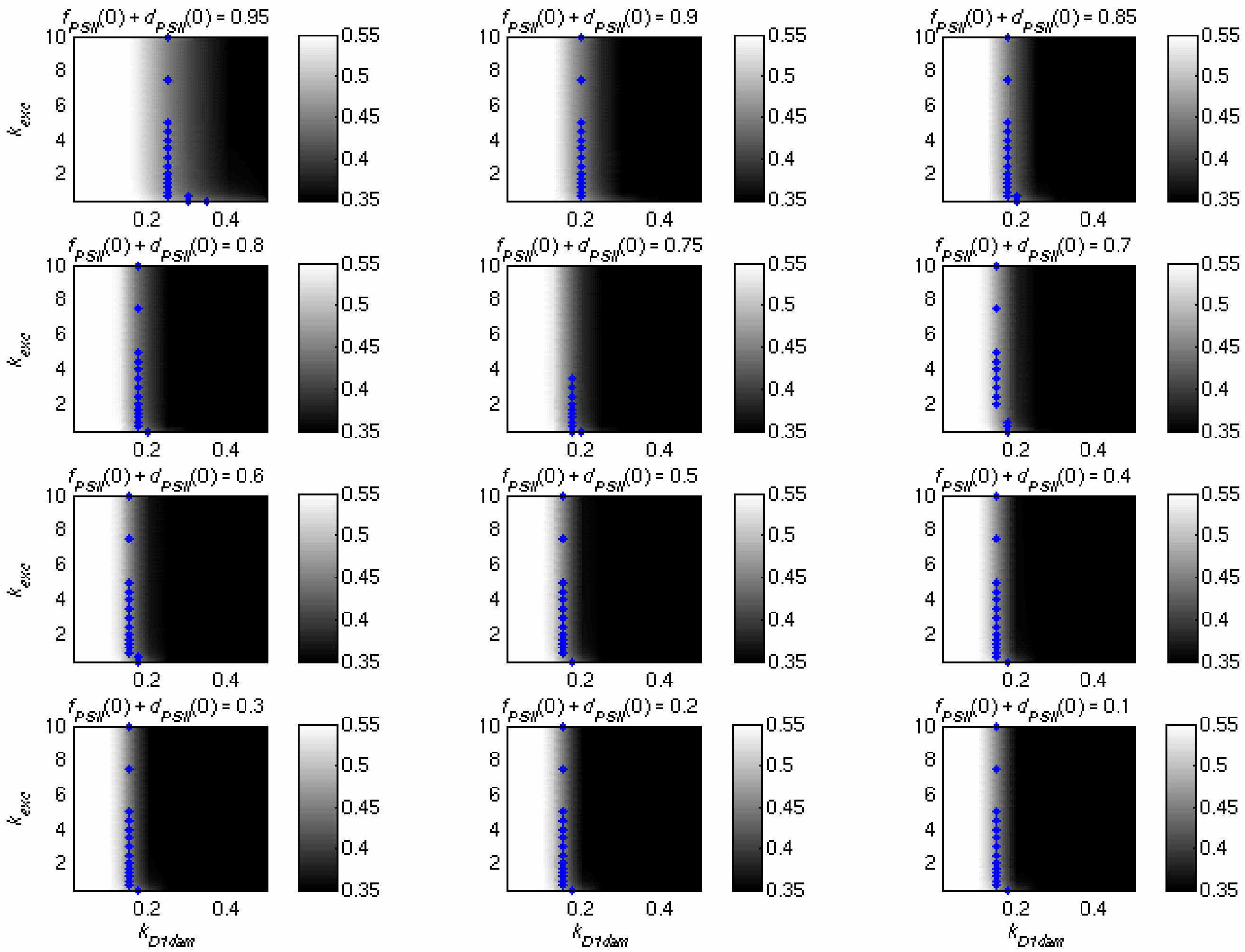

Fig. S2. As for Fig. S1, but a higher rate of *psbA* mRNA decay ($d_{RpsbA} = 0.72$).

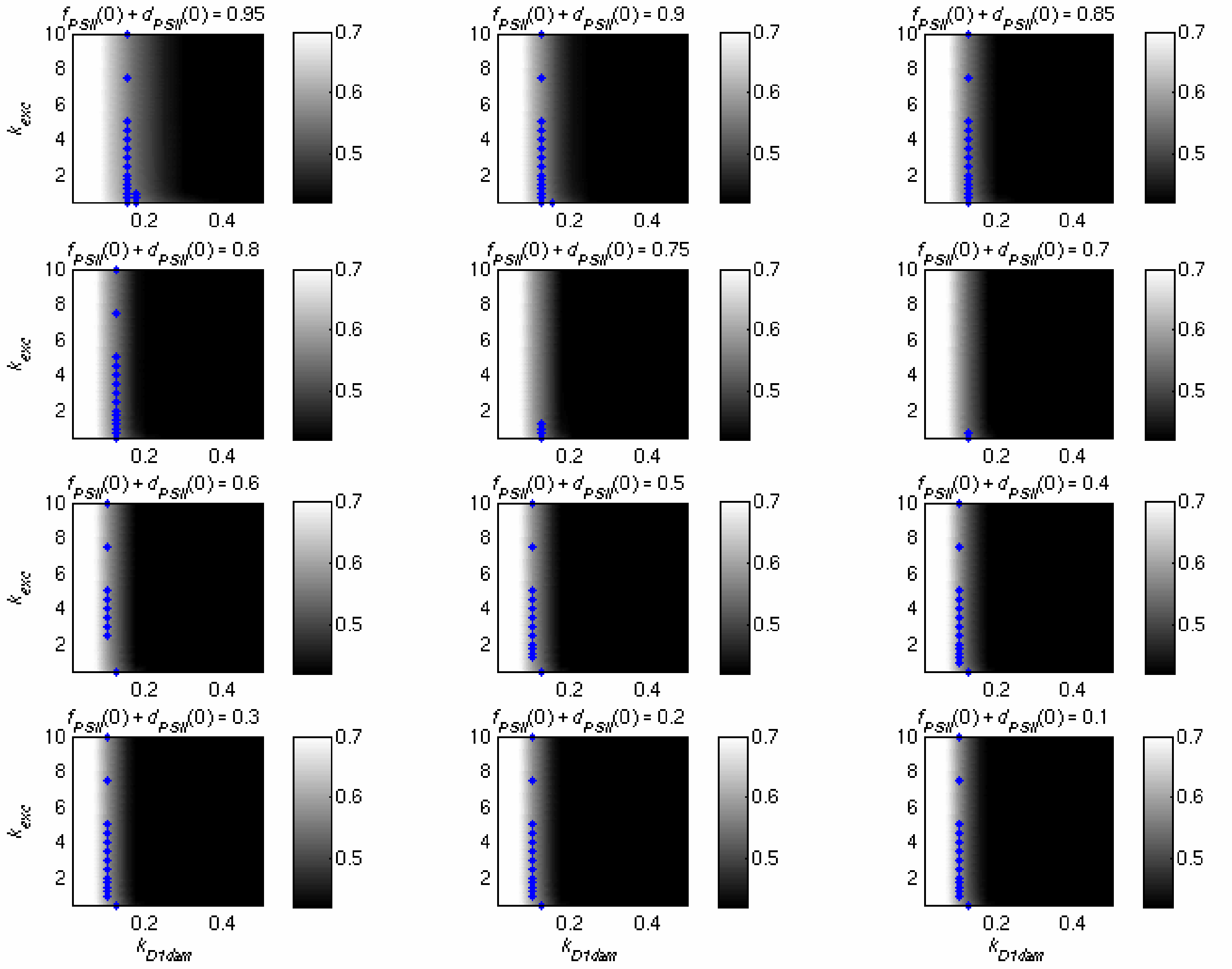

Fig. S3. As for Fig. S1, but with a higher rate of *psbA* mRNA decay (d_{RpsbA} = 0.72), and assuming that 63% of cells were infected in experiments where phage *psbA* expression was measured [3]. Here, blue dots illustrate combinations of parameter values that can account for the drop in levels of host D1 protein to 56% of their initial value, following 8 hours of infection (±5%), in the presence of appropriate expression of *psbA* by phage.

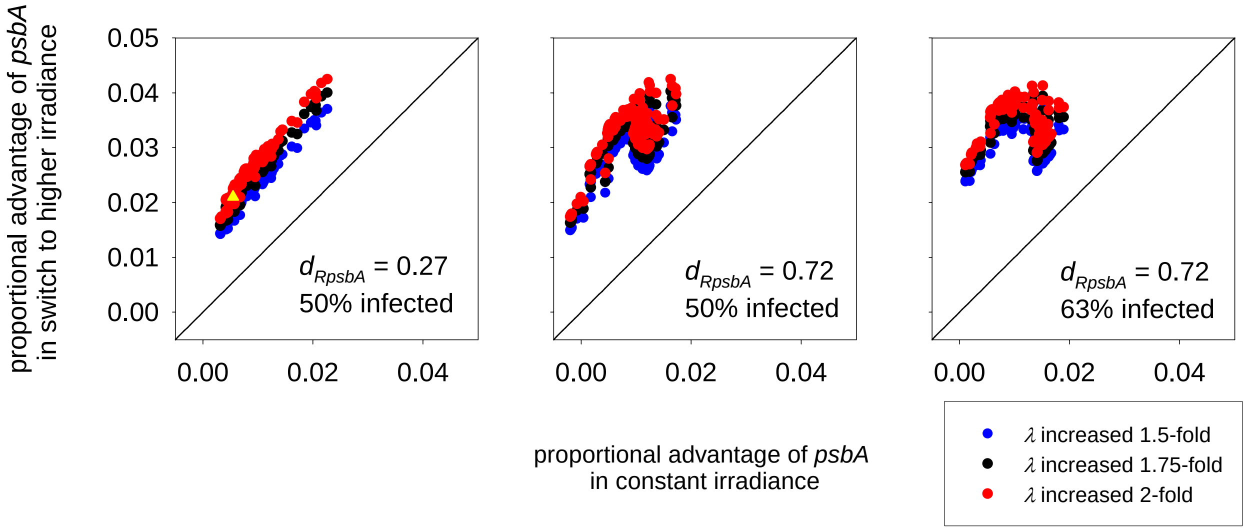

Fig. S4. The proportional advantage of phage encoded *psbA* to phage genome replication when irradiance is 25 μE m^{-2} s^{-1} and constant (horizontal axis) versus under conditions when irradiance is increased from 25 μE m^{-2} s^{-1} to 50 μE m^{-2} s^{-1} one hour following infection (vertical axis). Here we assume that the phage produced extra dNTPs in the light from a source other than the host genome (i.e., $\lambda > 0$ and $\kappa = 0$). The three panels show results for different combinations of d_{RpsbA} and the proportion of cells that were assumed to be infected. For each combination of k_{D1dam}, k_{exc}, and $x_{PSII}(0)$ values (those illustrated in blue in Figs S1-S3), we performed simulations of phage genome replication for a 'wild type' phage, and for a 'mutant' phage that does not express or encode the *psbA* gene. We then calculated the proportional advantage of encoding *psbA* as the difference between the number of genomes in cells infected by the wild type (G_W) and mutant (G_M) phages after 8 h, expressed relative to the number of genomes in a cell infected by the wild type (*i.e.*, $(G_W - G_M) / G_W$). We calculated this proportional advantage in constant irradiance of 25 μE m^{-2} s^{-1}, and when irradiance is increased from 25 μE m^{-2} s^{-1} to 50 μE m^{-2} s^{-1} one hour after infection. We performed these analyses for each of three different possible outcomes of the increase in irradiance: a 1.5-fold (blue dots), 1.75-fold (black dots) and 2-fold (red dots) increase in the rate of photosynthesis (which is included in λ). We see that *psbA* confers a greater proportional advantage to phage replication when irradiance increases, particularly when the increase in irradiance has a large (2-fold) effect on photosynthetic rate. The yellow triangle corresponds to the simulation that was considered in detail in the paper: $k_{D1dam} = 0.35$, $k_{exc} = 4$, and $x_{PSII}(0) = 0.5$, and with a 1.75 increase in photosynthesis following the increase in irradiance. (Note that these analyses use different values of k_{RPpsbA} and λ. For each combination of k_{D1dam}, k_{exc}, and $x_{PSII}(0)$, we identified a value of k_{RPpsbA} that could account for the observation that phage D1 comprised 10% of total D1 protein after 8 hours of infection (±2.5%; [3]), and a value of λ that could account for the observed [3] number of phage genomes made per cell in the light after 8 hours of infection (±2.5%).

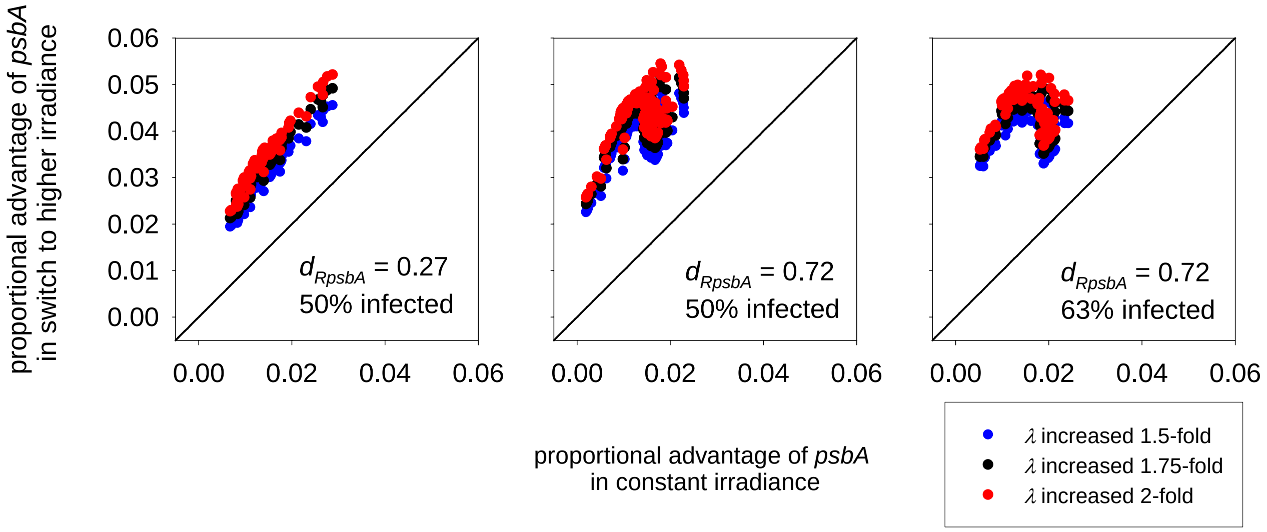

Fig. S5. As for Supplementary Fig. S4, except assuming that extra dNTPs produced by the phage in the light were made from the degraded host genome. Here κ was determined using the approach described for λ in Fig. S4, while λ was set to zero.

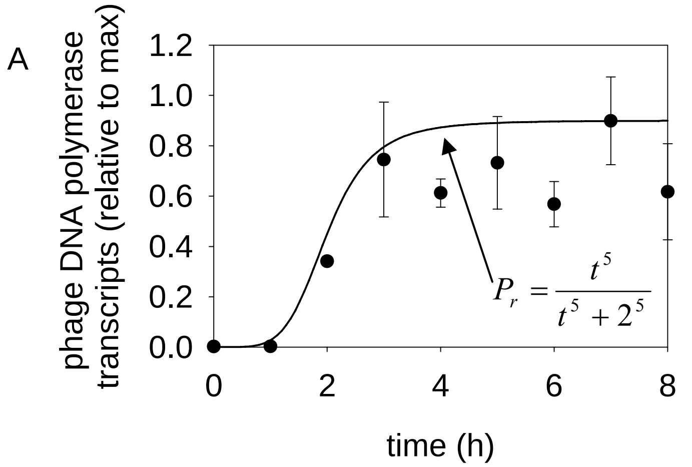

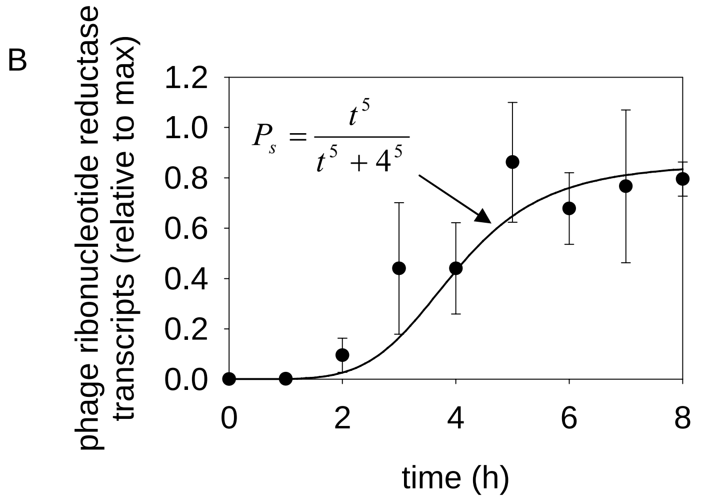

Fig. S6. Expression of phage (A) DNA polymerase and (B) ribonucleotide reductase mRNAs as a function of time since infection (data are means ± s.d.). Expression was measured by quantitative RT-PCR (relative to the gene *rnpB*), and reported by Lindell et al. [4]. Phage DNA polymerase is likely necessary for phage genome replication and ribonucleotide reductase is believed to be involved in the production of dNTPs. Fitted lines illustrate the functions used in our model for (A) DNA replication (P_r, with $t_r = 2$) and (B) dNTP production by the synthesis of new deoxynucleotides (P_s, with $t_s = 4$), normalized to reach 1 at the maximum mean measured level of expression.

References

1. Tyystjärvi E, Mäenpää P, Aro E-M (1994) Mathematical modelling of photoinhibition and photosystem II repair cycle. I. Photoinhibition and D1 protein degradation *in vitro* and in the absence of chloroplast protein synthesis *in vivo*. Photosynthesis Research 41: 439-449.
2. Six C, Finkel ZV, Irwin AJ, Campbell DA (2008) Light variability illuminates niche-partitioning among marine picocyanobacteria. PLoS One 2: e1341.
3. Lindell D, Jaffe JD, Johnson ZI, Church GM, Chisholm SW (2005) Photosynthesis genes in marine viruses yield proteins during host infection. Nature 438: 86-89.
4. Lindell D, Jaffe JD, Coleman ML, Futschik ME, Axmann IM, et al. (2007) Genome-wide expression dynamics of a marine virus and its host reveal features of co-evolution. Nature 449: 83-86.
